# Epidemiological investigation of Senecavirus A infection in pig herds in China from 2018 to 2021

**DOI:** 10.3389/fvets.2024.1391513

**Published:** 2024-07-01

**Authors:** Chenyu Li, Chunliu Gao, Longfei Tao, Jin Cui, Hui Zhang, Hui Zheng, Rong Wei, Shaopeng Gu, Zhou Sha, Bo Ni

**Affiliations:** ^1^College of Veterinary Medicine, Shanxi Agricultural University, Jinzhong, China; ^2^China Animal Health and Epidemiology Center, Qingdao, China; ^3^New Hope Liuhe Co., Ltd., Chengdu, China

**Keywords:** SVA, pig populations, epidemiological study, individual positive rate, herd positive rate

## Abstract

Senecaviurs A (SVA) infection, an emerging infectious disease in pig populations, is characterized by vesicular lesions predominantly affecting the mouth, snout, and hooves of infected pigs, similar to the symptoms of Foot and Mouth Disease Virus (FMDV). This disease first spread into China in 2015, causing great panic in the pig breeding industry. To determine the prevalence of SVA in pig herds in China from 2018 to 2021, a total of 4,901 pig tissue samples were collected from 18 provinces, autonomous regions and municipalities (P.A.M.s) for epidemiological investigation, virus isolation and genetic analysis. In 2021, the individual positive rates (IPRs) from the perspective of spatial distribution in East China, South China, Central China, North China, Southwest China, Northwest China, and Northeast China were 0, 0, 1.69, 0.94, 11.70, 3.31 and 2.21%, respectively. The herd positive rates (HPRs) were 0, 0, 9.52, 9.09, 50.00, 7.69 and 23.08%. From the perspective of temporal distribution, the IPR showed an overall downwards trend from 2018 to 2021, with only a slight increase in 2020. Moreover, the HPR decreased from 36.63 to 10.07%. From the perspective of population distribution in 2021, the IPR (2.62%) and HPR (12.00%) in apparently healthy pig herds (slaughterhouses) were greater than those in non-healthy pig herds (2.10 and 5.13%, respectively), consistent with the results in 2019. To characterize the prevalent strains, 10 SVA strains isolated from positive samples in 2019 were clustered in Clades I and VII; SVA-FJ039-2019, SVA-HuN032-2019, SVA-GX011-2019, SVA-FJ036-2019, SVA-GXF011-2019 and SVA-GXF053-2019 were clustered in Clade I; and SVA-FJ018-2019, SVA-SD069-2019, SVA-SD072-2019, and SVA-SD074-2019 were clustered in Clade VII. In conclusion, until 2021, the prevalence of SVA in pig herds in China was still relatively high, the contaminated area was still large, and there were a number of hidden infections. In the future, the epidemic status of SVA in pig herds in China must be closely monitored and the prevention and control measures must be adjusted in a timely manner.

## 1 Introduction

Senecavirus A (SVA) belongs to the genus *Senecavirus* in the family *Picornaviridae*. It is the only SVA serotype that has been identified (1). The virus was first discovered in a human embryonic retinal cell line (PRE. C6) by American researchers in 2002 and was named SVV-001 (2). The viral particle has a diameter of 25–30 nm and exhibits an icosahedral structure with no envelope (3). It is a single-stranded positive-sense RNA virus with a genome length of approximately 7300 nt, featuring a typical “L-4-3-4” structure of the small RNA virus family (4).

SVA can infect pig herds of different types and ages. Reports have confirmed that SVA is associated with porcine idiopathic vesicular disease (PIVD) and increased mortality in newborn piglets (5, 6). After infection, piglets, finishing pigs, and multiparous sows may experience ulcers in the nasal and oral cavities, as well as red blisters in the nose. Additional clinical symptoms may include occasional fever, lameness, and diarrhea. Acute death can occur in some cases (7). Notably, SVA shows similar transmission characteristics and clinical symptoms to Foot and Mouth Disease Virus (FMDV), and it is difficult to differentiate these two diseases with clinical diagnosis (2). Currently, neither vaccines nor drugs are commercially available for preventing SVA. Expensive laboratory diagnostics, especially qRT–PCR and ELISA, are required for diagnosis, making the prevention and control of SVA significantly challenging (5).

The first outbreak of SVA occurred in Canada in 2007. Furthermore, it has spread to multiple countries worldwide, including Brazil, Canada, the United States, and Thailand, and has become a globally prevalent virus infecting pigs (8). SVA first appeared in Guangdong, China, in March 2015 (9). Subsequently, outbreaks of SVA were reported in Fujian, Henan, and other regions from 2016 to 2017 (10). Ding et al. (11) reported a significant epidemiological correlation between successive SVA outbreaks that occurred in Guangdong and Hubei Provinces during retrospective monitoring of the SVA epidemic from 2015 to 2016. Zhang et al. (12) conducted retrospective monitoring of porcine SVA infection in seven provinces in China, including Liaoning and Fujian Provinces, as early as 2016. Since then, many epidemiological investigations have been conducted, but the majority of these studies are limited to local areas. In this study, we conducted an epidemiological study in 18 P.A.M.s of China from 2018 to 2021 to investigate the prevalence of SVA in China. Additionally, a phylogenetic analysis of 12 SVA strains was also conducted to analyse the prevalent strains (12).

## 2 Methods and materials

### 2.1 Experimental design and sample collection

An epidemiological investigation was designed and conducted to investigate the prevalence of SVA in Chinese pig herds. In 2018, only 8 primary pig-producing provinces, autonomous regions and municipalities (P.A.M.s) were investigated. As the area contaminated with SVA progressively expanded, the number of P.A.M.s included in the investigation gradually increased and reached 15 by 2021 (Table 1). The samples were collected from two types of pig herds: samples from slaughterhouses representing healthy pig herds and samples from sick pigs and dead pigs (not specifically caused by SVA infection) from non-healthy pig farms representing non-healthy pig herds. The samples were collected by the veterinary authorities of the respective P.A.M.s and then sent to the China Animal Health and Epidemiology Center (CAHEC) for further analysis. A total of 4,901 tissue samples (each from the tonsils, spleen, kidneys, lymph nodes, lungs, etc., from one pig) were collected from 277 slaughterhouses and 95 non-healthy pig farms in 18 P.A.M.s (Table 1).

**Table 1:** Sampling information of SVA.

	2018			2019			2020			2021		
	P.A.M.s	Pig herds	Tissue sample	P.A.M.s	Pig herds	Tissue sample	P.A.M.s	Pig herds	Tissue sample	P.A.M.s	Pig herds	Tissue sample
Slaughterhouses	Xinjiang, Guangxi, Fujian, Shandong, Liaoning, Hunan, Hubei	35	553	Xinjiang, Guangxi, Fujian, Shandong, Liaoning, Hunan, Hebei, Tianjin, Heilongjiang, Guangdong	76	1,063	Guangxi, Fujian, Liaoning, Hunan, Hebei, Tianjin, Heilongjiang, Guangdong, Henan, Shaanxi, Guizhou, Yunnan	66	1,228	Xinjiang, Guangxi, Fujian, Shandong, Liaoning, Hunan, Hebei, Tianjin, Heilongjiang, Guangdong, Henan, Shaanxi, Guizhou, Yunnan, Beijing	100	1,414
Non-healthy pig farms	Liaoning, Sichuan, Shandong	5	84	Guangxi, Jilin, Shandong, Henan, Shaanxi	16	247	Guangxi, Shandong, Henan, Shaanxi	35	169	Guangxi, Henan, Shaanxi	39	143

### 2.2 Tissue sample processing

The tissue samples were resuspended in 1 mL of PBS and then ground for 10 min. Subsequently, centrifugation was performed at 12,000 rpm for 10 min by utilizing a high-speed centrifuge at 4°C. The supernatant was used for RNA extraction following the instructions provided with the magnetic bead virus RNA/DNA extraction kit (Xi’an Tianlong Science and Technology Co., Ltd.).

### 2.3 qRT–PCR

qRT–PCR was performed using the following primer sequences: SVA-F, 5’-CTGCGCTGGGACCGTATCTCA-3′; SVA-R, 5’-CGCCCCACCCTCATT-3′; and SVA-P, 5’-FAM-TCGCCCGTAAGCGCGCACCGAGAG-BHQ1. The amplification program for qRT–PCR was as follows: reverse transcription at 55°C for 15 min, predenaturation at 95°C for 30 s, denaturation at 95°C for 10 s, and annealing and extension at 60°C for 30 s for a total of 45 cycles. The judgment criteria for the results were as follows: Ct < 35 was considered to be positive, 35 ≤ Ct ≤ 37 was considered suspected, and Ct > 37 was regarded as negative. The suspected samples were confirmed before being recorded as positive. The suspected or negative results after confirmation were recorded as negative.

### 2.4 SVA isolation

SVA was isolated from the BHK-21 cell line (stored in CAHEC) using Dulbecco’s modified Eagle’s medium (Vivacell, #C3113-0500) supplemented with 2% fetal bovine serum (Opcel, #BS-1101) and 1% penicillin–streptomycin (Vivacell. #C3423-0100). Ten distinct SVA strains were successfully isolated from qRT–PCR-positive samples.

### 2.5 SVA fragment RT–PCR amplification

The nucleic acid samples from the 2019 samples were reverse transcribed by HiScript® III RT Supermix for qPCR (+cDNA Wiper), after which the whole-genome sequence of SVA was amplified in 7 segments (Table 2) by using Phanta® Max as a template. The amplification program was as follows: predenaturation at 94°C for 2 min, 30 cycles of denaturation at 94°C for 30 s, annealing at 55°C for 30 s, and extension at 72°C for 1 min. After the reaction, the PCR products were identified and detected by 1% agarose gel electrophoresis; the PCR products were subsequently sent to Shenggong Bioengineering (Qingdao) Co., Ltd., for sequencing and sequence assembly.

**Table 2:** Primers for amplification of the full-length SVA genome.

Primer name	Primer sequence 5′–3’
1F	TTTGAAATGGGGGGCTGG
1R	TGGCAGTAAAAGTGGTGGTGGGT
2F	CGGATTAGCGGGTCTCCTCACA
2R	CGAACTGGGCAGGAACAGCAACG
3F	ACCTCTTACAACTGGCCCGTATA
3R	GTTCCAAGGGAGCACGAAAGATA
4F	GGCAGTGAGTACCAGGCTTCTA
4R	CACTTTGTGAGCCATAGAGACGC
5F	CCCAAAGTCTCACCACTATGATC
5R	TAGATTGTTAGGGAAAGAGTTGC
6F	AAGTGGTCGTCCCCATTACCTT
6R	ACGGTCAGAATGTCATTGTCTTTTC
7F	GTGTTGTCAAAATTTGATCCCAGAC
7R	CTTTTCTGTTCCGACTGGGTTCT

### 2.6 Data processing

Due to the inconsistency in the P.A.M.s where samples are collected each year, we conducted a prevalence analysis based on seven geographical regions of China, namely, East China (Shandong Province, Fujian Province, etc.), South China (Guangdong Province, Guangxi Zhuang Autonomous Region, etc.), Central China (Henan Province, Hunan Province, Hubei Province, etc.), North China (Tianjin, Beijing, Hebei Province, Inner Mongolia Autonomous Region, etc.), Southwest China (Yunnan Province, Guizhou Province, etc.), Northwest China (Xinjiang Uygur Autonomous Region, Ningxia Hui Autonomous Region, Gansu Province, Shaanxi Province, etc.) and Northeast China (Heilongjiang Province, Liaoning Province, etc.).

### 2.7 SVA genome assembly and phylogenetic analysis

SVA genome assembly was carried out by SeqMan, and the 7-segment sequences of each strain were assembled into a whole-genome sequence. Ten isolated SVA sequences and 157 reference SVA sequences from eight clades were included as previously reported (13). The Neighbor-Joining (NJ) tree was applied to the SVA sequences using Mega 11. The homology analysis was performed with Megalign.

## 3 Results

### 3.1 Spatial distribution

**Figure 1:**
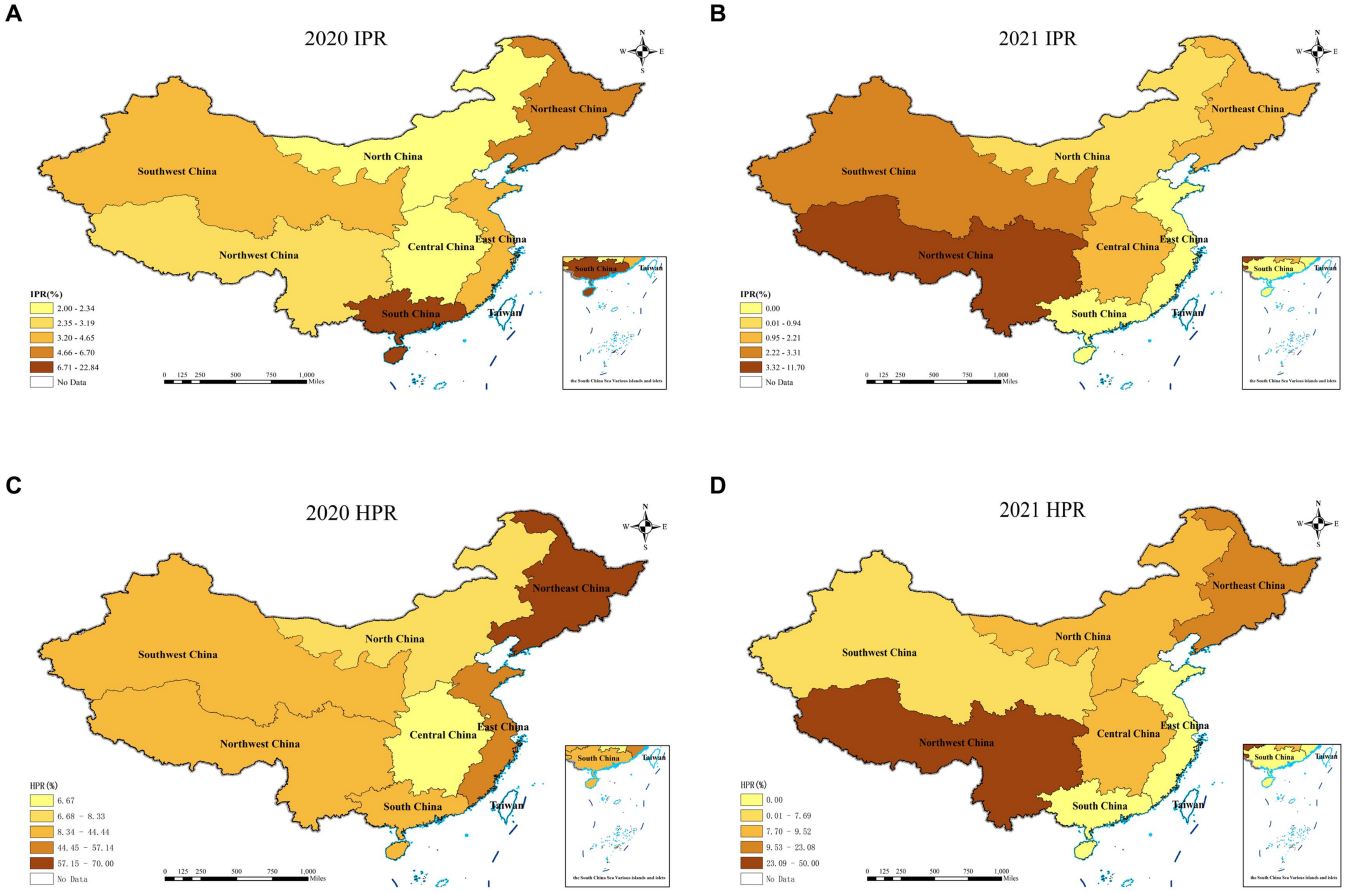
Individual positive rates and herd positive rates of SVA detected in each region from 2020 to 2021.

In 2021, the overall individual positive rate (IPR) in P.A.M.s was 2.57%, while the herd positive rate (HPR) was 10.07%. The IPRs in East China, South China, Central China, North China, Southwest China, Northwest China, and Northeast China were 0, 0, 1.69, 0.94, 11.70, 3.31 and 2.21%, respectively. The HPR of Southwest China reached 50.00%. Northwest China had a positive rate of 7.69%, followed by Central China with 9.52%, North China with 9.09%, and Northeast China with 23.08%. No SVA-positive samples were detected in East China or South China (Figure 1).

### 3.2 Temporal distribution

**Figure 2:**
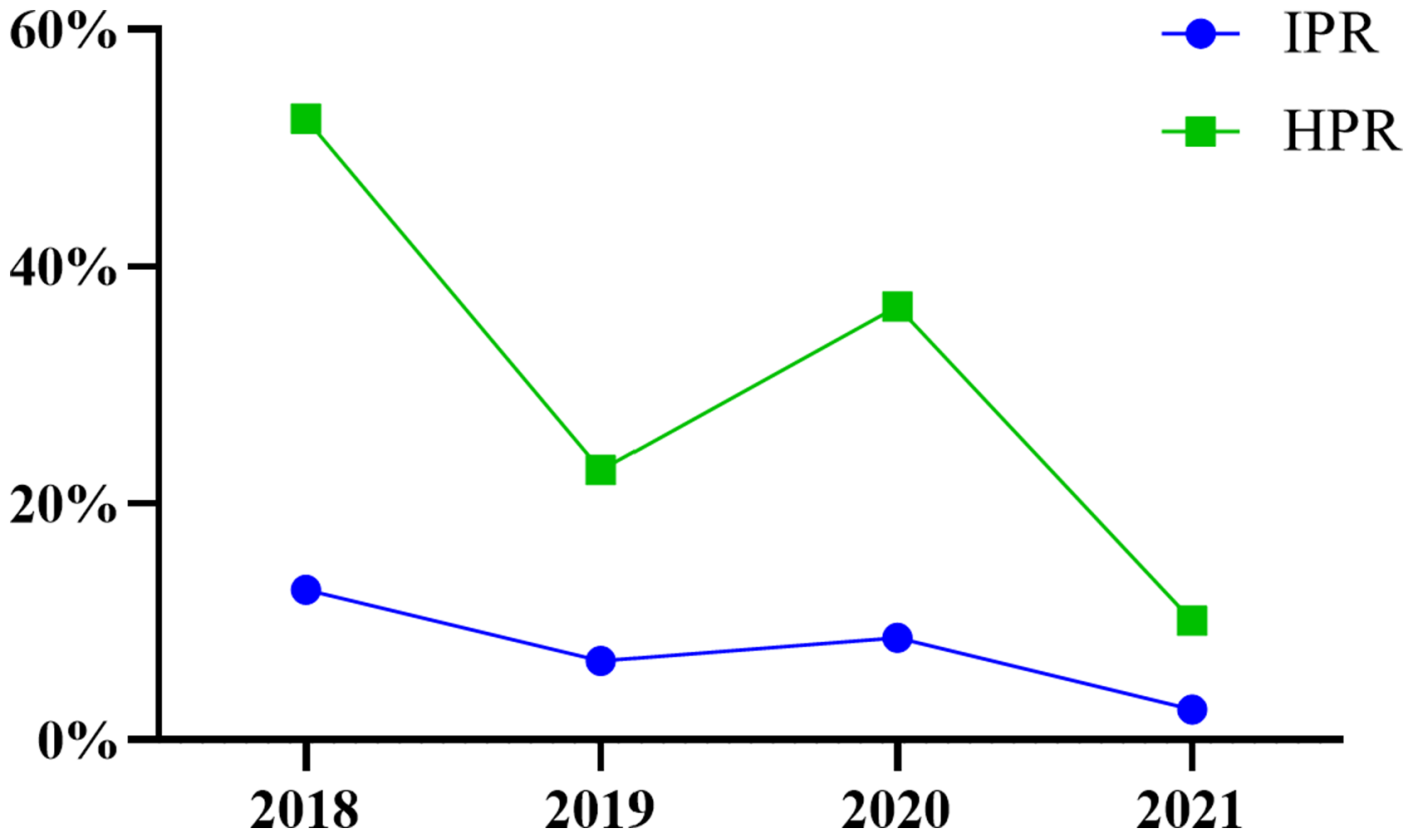
Temporal distribution of SVA in 2018–2021.

The IPR and HPR of the SVA exhibited a fluctuating declining trend from 2018 to 2021 in China (Table 3; Figure 2). Specifically, compared to 2020, the IPR decreased from 8.66 to 2.57%, and the HPR decreased from 36.63 to 10.07%. These findings suggested a notable decline in the prevalence of SVA from 2018 to 2021. When analysing the IPRs and HPRs in different regions of China (Figure 1), the IPRs in the seven regions in 2021 exhibited varying trends compared to those in 2020. Among these regions, the majority (6 out of 7) showed a decrease in the IPR, except for Southwest China. Similarly, the HPRs also demonstrated a downwards trend in 4 out of 7 regions, but an increasing trend was observed in the HPRs in North China, Central China, and Southwest China in 2021.

**Table 3:** Temporal distribution of SVA in 2018–2021.

Year	Number of positive samples/total samples	Individual positive rate	Positive herd number/total herd number	Herd positive rate/%
2018	81/637	12.70%	21/40	52.50%
2019	88/1310	6.72%	21/92	22.83%
2020	121/1397	8.66%	37/101	36.63%
2021	40/1557	2.57%	14/139	10.07%

### 3.3 Population distribution

**Figure 3:**
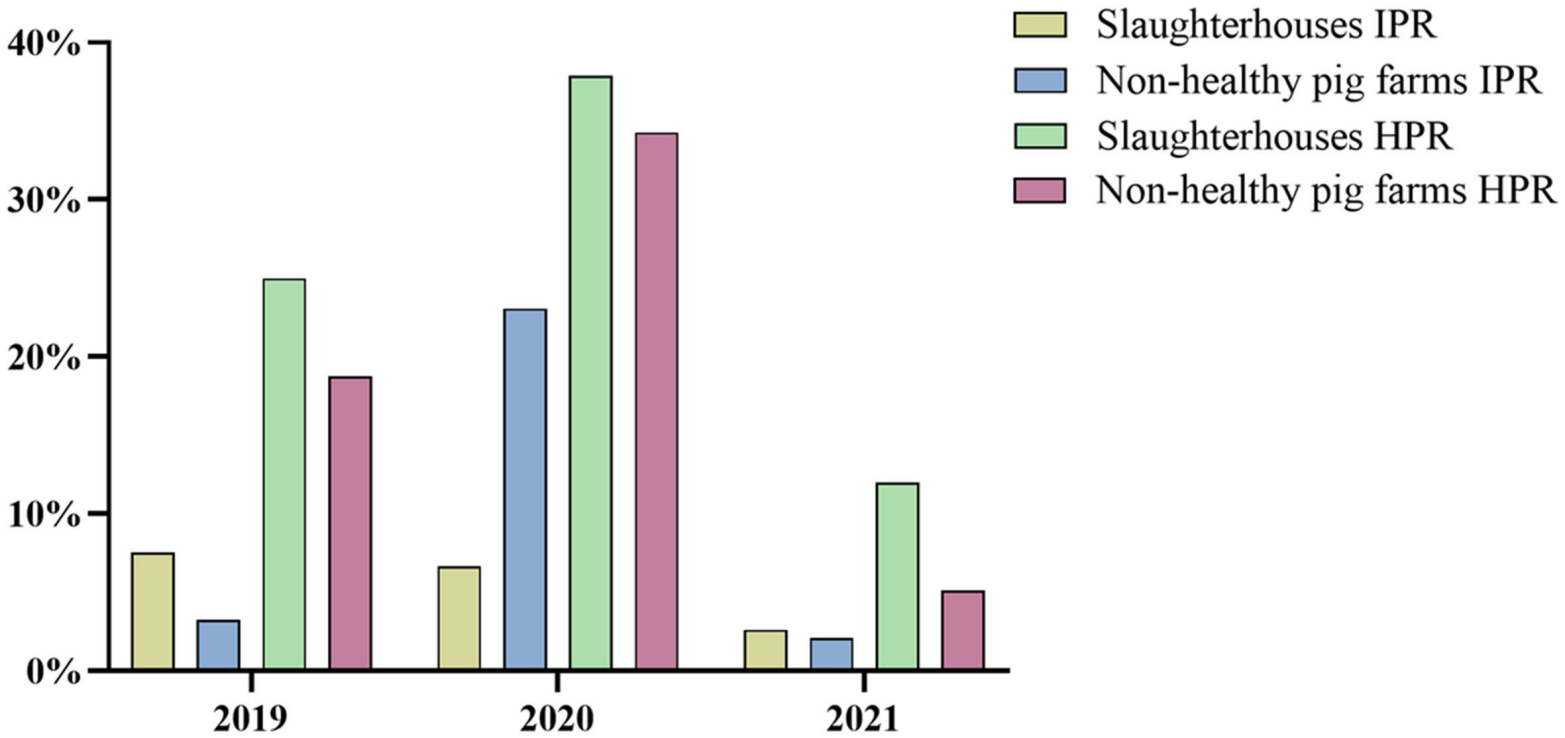
Comparison of IPR and HPR between slaughterhouse and non-healthy pig farms from 2019 to 2021.

As shown in Figure 3, the IPR in slaughterhouses in 2021 was 2.62%, while in non-healthy pig farms, it was slightly lower at 2.10%. Moreover, the HPR in slaughterhouses in 2021 was 12.00%, which was higher than the 5.13% observed in non-healthy pig farms. These results were consistent with the data from 2019, in which the slaughterhouses exhibited greater IPRs and HPRs than did non-healthy pig farms. However, there was a change in this trend in 2020 for the IPR. During this year, the incidence of SVA was greater in non-healthy pig farms than in slaughterhouses.

### 3.4 Genetic evolution virus isolation and homology analysis

**Figure 4:**
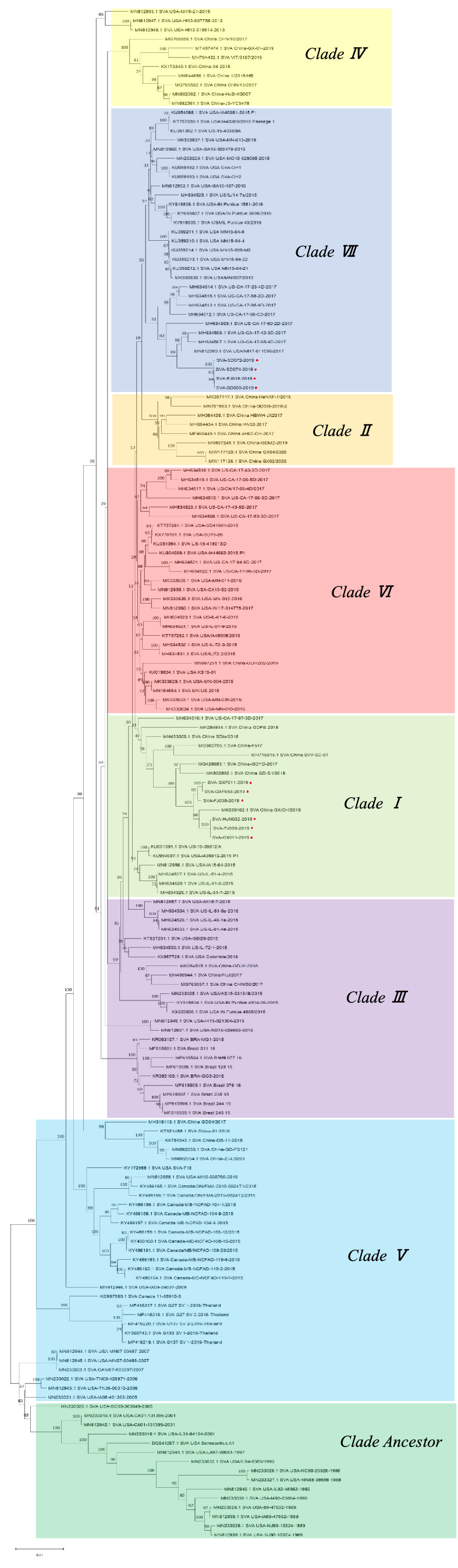
Phylogenetic tree of 10 SVA isolates.

Ten SVA strains isolated from SVA-positive samples in 2019 were sequenced, and phylogenetic tree analysis was conducted using the Neighbor-Joining (NJ) method in MEGA11. Ten SVA isolates were clustered in Clade I or Clade VII. SVA-FJ018-2019, SVA-SD069-2019, SVA-SD072-2019, and SVA-SD074-2019 were clustered in Clade VII, showing a genetic association with the USA/MI17-011956/2017 strain, which was identified in the United States. Additionally, the isolates SVA-FJ039-2019, SVA-HuN032-2019, SVA-GX011-2019, SVA-FJ036-2019, SVA-GXF011-2019 and SVA-GXF053-2019 were clustered in Clade I (Figure 4).

**Figure 5:**
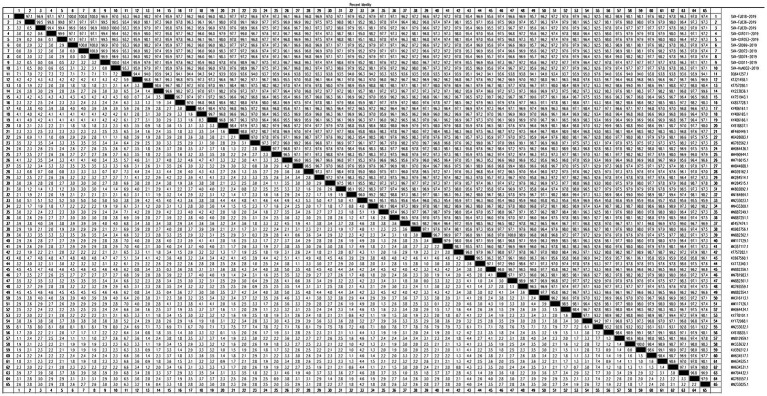
Homology analysis of 10 SVA isolates.

The homology analysis was performed with Megalign. The percentages of isolates SVA-FJ018-2019, SVA-SD069-2019, SVA-SD072-2019, SVA-SD074-2019, and the American isolate MI17-011956/2017 were approximately 98.90, 98.90, 98.90, and 99.00%, respectively. SVA-GXF011-2019, SVA-GXF053-2019, SVA-FJ036-2019, SVA-FJ039-2019, SVA-GX011-2019, and SVA-HuN032-2019 had the highest homology with the Chinese strain SVA/GX/CH/2018, ranging from 99.20 to 99.30% (Figure 5).

## 4 Discussion

The first SVA case was found in Guangdong Province, China, in 2015, which raised considerable concerns in the pig industry. Since then, a series of epidemiological studies on SVA have been performed in China. However, most of these investigations were disparate and localized, and a comprehensive epidemiological survey was lacking (14). To address this issue, an epidemiological survey was conducted in 18 P.A.M.s of China from 2018 to 2019. A total of 4,901 tissue samples were collected from 293 slaughterhouses and 104 pig farms. Due to variations in sampling across different years, it is more convenient to analyse the prevalence of SVA based on 7 geographical regions of China.

In our study, the IPRs of SVA in different regions varied significantly, ranging from as low as 0 to 11.70%. Notably, the IPR of SVA in Southwest China exceeded 10.00%, indicating a comparatively severe infection outbreak in this area. For the HPR, which was even more variable, spanning from 0% to a striking 50.00%, Southwest China once again showed the highest prevalence at 50.00%, while Northeast China experienced a lower but still concerning rate of 23.08%. These findings suggest that SVA contamination is widespread and particularly severe in some regions. Integrating our results with previous epidemiological studies conducted in the provinces of Hubei, Henan, and Hainan (15–20), it becomes apparent that a number of provinces across China have encountered SVA outbreaks. The temporal distribution of the SVA data between 2018 and 2021 reveals a fluctuating downwards trend in both the IPR and HPR. We suspected that this may be associated with the outbreak of African swine fever virus (ASFV) in China, after which national-level policies for prevention and control were introduced and effectively implemented, leading to a continuous decrease in the transmission of various epidemic diseases (21). The structure of pig breeding has gradually shifted from traditional small-scale household operations to large-scale farms, and the level of biosecurity protection in pig farms has continuously increased. This improvement not only helped in controlling ASFV (22) but also effectively prevented other epidemic diseases, such as SVA. However, we found that there was a sudden increase in the positivity rate observed in 2020, which we believe is primarily due to the rapid escalation of the pig herd population in China. In 2020, there was a significant increase in the number of reproductive sows and the total pig population, with year-on-year increases of 35 and 31%, respectively, compared to 2019. This increase implies greater movements within the pig population and higher stocking densities, contributing to an increased incidence of infectious diseases in pigs. Zhang et al. (23) carried out an epidemiological investigation of SVA prevalence in pig populations across Zhejiang Province. Their findings indicated fluctuating rates for SVA between 2018 and 2020, consistent with the trends observed in our research. The results for the population distribution indicated that there was a greater IPR and HPR in pigs from slaughterhouses than in those from non-healthy pig farms during the period from 2019–2021. Therefore, the health condition of the pig population is not the primary factor affecting SVA infection, and SVA should be transmitted and infected during the transportation process and preslaughter husbandry practices. Since SVA is prevalent in apparently healthy pig herds, there might be a subclinical infection of this virus in pig herds. Compared to the results of tissue sample detection, the IPRs and HPRs identified through serological testing in Northwest China are both much greater (data not shown), implying that the actual scope of SVA infection among pig populations might be broader than what tissue-based diagnostics alone have revealed.

Previous reports have indicated the presence of Clade Ancestor and Clade I-VII isolates in China (13). In this study, 10 epidemic strains were successfully isolated; the isolates SVA-FJ018-2019, SVA-SD069-2019, SVA-SD072-2019, and SVA-SD074-2019 were clustered in Clade VII, which was closely related to the American strain USA/MI17-011956/2017, suggesting the potential introduction of this strain through international trade, and further research is needed. Genomic sequencing has revealed marginal variance, with only approximately 20 amino acid variations, between attenuated and pathogenic strains of SVA (24). Given the potential for ongoing genetic evolution to enhance the virulence of prevalent strains of SVA in China, it is imperative to maintain vigilant monitoring and surveillance of SVA infection (25) and to develop accurate and specific differential diagnostic techniques to reduce the impact of SVA on the pig industry.

## 5 Conclusion

In conclusion, we conducted an epidemiological investigation of SVA in China from 2018 to 2021 to explore the prevalence of SVA in China. This epidemiological investigation provides a foundation for implementing effective SVA prevention and control measures to ultimately curb its propagation.

## Data availability statement

The datasets presented in this study can be found in online repositories (https://www.ncbi.nlm.nih.gov/genbank/). The accession numbers are as follows: SVA-FJ018-2019, PP888073; SVA-FJ036-2019, PP888074; SVA-FJ039-2019, PP888075; SVA-GXF011-2019, PP888076; SVA-GXF053-2019, PP888077; SVA-SD069-2019, PP888078; SVA-SD072-2019, PP888079; SVA-SD074-2019, PP888080; SVA-GX011-2019, PP888081; SVA-HuN032-2019, PP888082.

## Author contributions

CL: Investigation, Writing – original draft. CG: Investigation, Writing – original draft. LT: Investigation, Writing – original draft. JC: Data curation, Writing – review & editing. HZha: Data curation, Writing – review & editing. HZhe:Resources, Writing – review & editing. RW: Supervision, Writing –review & editing. SG: Methodology, Supervision, Writing – review & editing. ZS: Supervision, Writing – review & editing. BN: Methodology, Project administration, Supervision, Writing – review & editing.
